# Comprehensive Analysis of the Immune Implication of TEX41 in Skin Cutaneous Melanoma

**DOI:** 10.1155/2021/2409820

**Published:** 2021-11-09

**Authors:** Zhi-yong Chen, Jie-qing Huang, Yu Zhu, Yong-song Chen, Xue-feng Yu

**Affiliations:** Department of Burns and Plastic, The Fuling Center Hospital of Chongqing City, Fuling, Chongqing, China

## Abstract

Enhancer RNAs (eRNAs), a subclass of noncoding RNAs from enhancers, have been demonstrated to exhibit important regulatory effects on the expressions of various genes. However, the role of eRNAs in skin cutaneous melanoma (SKCM) remained largely unclear. In this study, we aimed to explore the expression and prognostic value of an enhancer RNA TEX41 in SKCM as well as the associations between TEX41 and tumor-infiltrating immune cells (TICs). We observed that TEX41 expression was distinctly increased in SKCM specimens compared with normal skin specimens using GEPIA. Survival assays based on TGCA datasets revealed that patients with low TEX41 expressions displayed a longer overall survival than those with high TEX41 expression. CIBERSORT datasets revealed that TEX41 was related to 8 types of TICs (macrophages M1, T cells regulatory, plasma cells, mast cells resting, T cells CD8, dendritic cells resting, and T cells follicular helper). Three kinds of TICs were negatively related to TEX41 expressions, including macrophages M2, NK cells resting, and macrophages M0. The expressions of TEX41 were involved in five KEGG pathways, including transcriptional misregulation in cancer, SNARE interactions in vesicular transport, mitophagy-animal, melanoma, melanogenesis, and progesterone-mediated oocyte maturation. Overall, TEX41 can be used as a novel biomarker for the prognosis of SKCM patients and is associated with TICs, indicating it as a therapeutic target for SKCM.

## 1. Introduction

Skin cutaneous melanoma (SKCM) accounts for only 3% of total skin tumors [1]. According to clinical statistics, SKCM resulted in >75 of deaths in skin tumors because of its metastatic abilities [2]. Based on the Clark model, the progression of SKCM from melanocytes to malignant melanoma is involved in a number of important actions, including formation of banal nevi, melanoma in situ, and invasive melanoma [3, 4]. Although melanoma patients with early stage have achieved a favorable five-year survival, the survivals for melanoma with stage III-IV are rarely longer than one year [5, 6]. To date, a large number of studies have delved into the mechanisms involved in recurrences and metastasis; the tumor progression of SKCM remains largely unclear.

Intrinsic genes of tumor cells have been demonstrated to exhibit a regulatory function on the developments of SKCM [7]. Besides, infiltrating immune cells in the tumor microenvironment are also proved to be involved in the regulation of the expressions of various tumor-related genes, thus exhibiting a potential modulation in the clinical prognosis of tumor patients [8, 9]. The SKCM microenvironment is generally immunosuppressive and contains infiltrating immune cells, including neutrophils, NK cells, macrophages, and microglia, but a paucity of T cells and nonimmune components [10, 11]. However, the potential mechanisms involved in the modulation of immune genes and immune cells associated with SKCM prognosis remained largely unclear. In recent years, more and more studies have demonstrated that the occurrences and developments of many types of diseases are associated with the dysregulation of noncoding RNAs [12, 13]. Among these, RNAs generated from enhancers (enhancer RNAs (eRNAs)) have attracted more and more attention, and more and studies have confirmed that eRNAs may act as novel biomarkers for diagnosis and prognosis of tumor patients and therapeutic targets and could mediate antioncogenic or tumor promotive functions in various tumors [14–16]. However, the potential function and effects of eRNAs in SKCM were rarely reported.

lncRNA testis expressed 41 (TEX41) was a newly identified tumor-related enhancer RNA [17]. Several studies have reported that TEX41 was dysregulated in several tumors, such as breast cancer, leukemia, and cervical cancer [18–20]. However, the specific function of TEX41 in tumors remained largely unclear. In addition, the clinical significance of TEX41 in SKCM patients and its association with infiltrating immune cells have not been investigated.

## 2. Methods and Materials

### 2.1. Collection of SKCM-Expressing Pattern Using TCGA Datasets

SKCM datasets were downloaded from TCGA datasets (https://portal.gdc.cancer.gov/). The SKCM datasets embraced 471 tumor specimens and 1 nontumor specimen which included the related clinical data. The expressing data of RNAs were processed by the use of the limma package for R software which used the voom functions. To determine the prognostic genes in SKCM, the R package “survival” was applied to study the associations between SKCM and the survival data using clinical data of SKCM in TCGA. GEPIA (http://gepia.cancer-pku.cn/) was applied for the determination of expressing pattern of TEX41. All original data are downloaded from TCGA datasets; thus, ethical approval and informed consent are unnecessary.

### 2.2. Determination of Tumor-Infiltrating Immune Cells (TICs) in TCGA SKCM

Our group used CIBERSORT methods to qualify 22 types of immune cells in all specimen samples [21]. By the use of the Monte Carlo sampling, our group measured an empirical *P* value for the deconvolution of every sample. After excluding samples with *P* ≥ 0.05, 471 SKCM samples and 1 normal sample were included for subsequent assays.

### 2.3. Functional Enrichment Analyses

To delve into the mechanisms involved in the effects of TEX41 on the prognosis of SKCM patients, Gene Ontology (GO) and Kyoto Encyclopedia of Genes and Genomes (KEGG) functional enrichment assays were carried out by the use of the R package (“clusterProfiler”) [22]. The top 10 biological process (BP), cellular component (CC), and molecular function (MF) GO terms and the top 30 KEGG pathways with an FDR < 0.05 were established as being significant by the use of R package (“ggplot2”) [23].

### 2.4. Statistical Analysis

Statistical analysis was performed in R software version 3.6.1 (The R Foundation, Vienna, Austria). The statistical significance in the basic characteristics was analyzed by the use of Pearson's chi-square test methods. Log-rank test was used to analyze the overall survival, followed by Kaplan-Meier which was used to plot survival curves. All tests were two tailed, and results with *P* < 0.05 were considered statistically significant.

## 3. Results

### 3.1. Increased Expressions of TEX41 and Its Prognostic Value in SKCM

**Figure 1:**
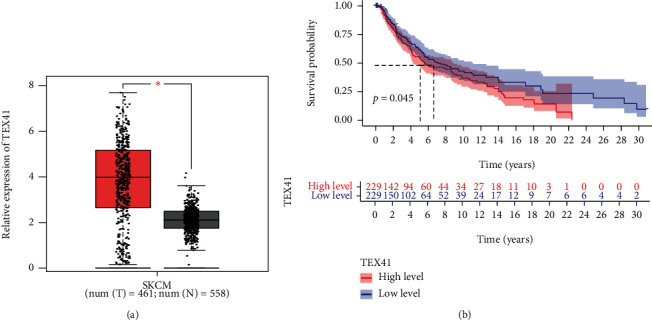
The expression of TEX41 in SKCM and its clinical significance. (a) The distinct upregulation of TEX41 was observed in SKCM analyzed by GEPIA. (b) Survival assays based on TCGA datasets. A poor prognosis was observed in SKCM patients with high TEX41 expression. ^∗^*P* < 0.05.

To explore the possible function of TEX41 in SKCM, we searched GEPIA and found that TEX41 expression was distinctly upregulated in SKCM specimens compared with normal skin specimens (*P* < 0.01, Figure 1(a)). Survival assays using TCGA datasets revealed that SKCM patients with high TEX41 expression displayed a shorter overall survival than those with low TEX41 expression (*P* = 0.045, Figure 1(b)).

### 3.2. The Landscape of Infiltrating Immune Cells in SKCM and Nontumor Specimens

**Figure 2:**
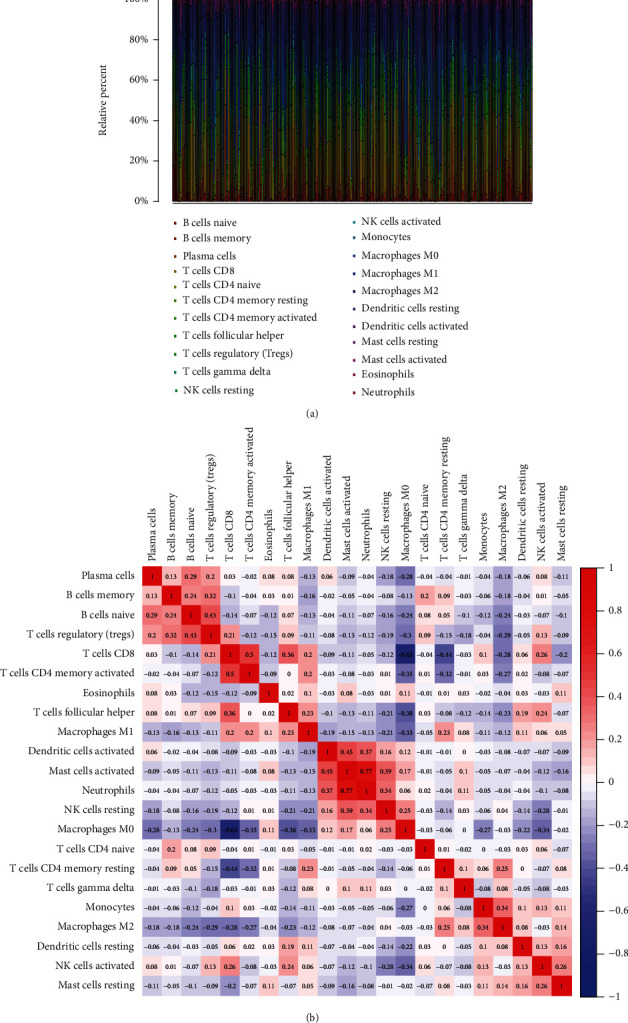
TIC profiles in SKCM specimens and correlation assays. (a) The bar chart summarized the percentage of 22 infiltrated immune cells from normal (*n* = 1) and SKCM (*n* = 471) specimens. (b) Heat map of 22 infiltrating immune cells in all samples.

**Figure 3:**
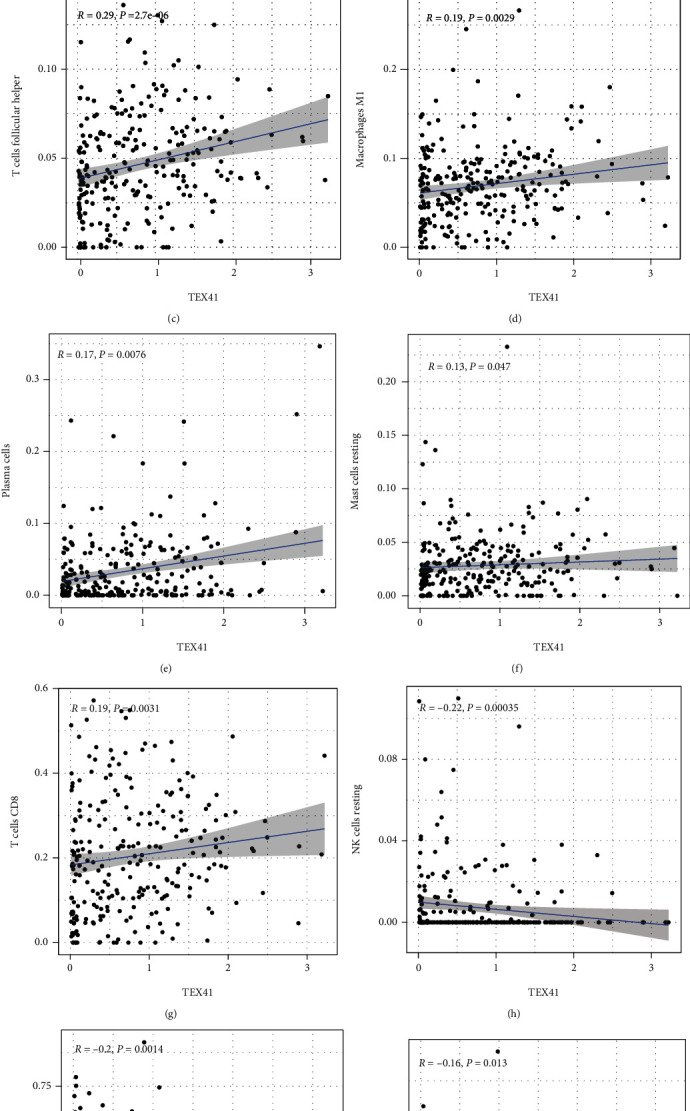
Association of TIC proportion with TEX41 expressions. Scatter plot showed the association of 10 kinds of TICs in proportion with the TEX41 expressions (*P* < 0.05), including (a) dendritic cells resting, (b) T cells regulatory (Tregs), (c) T cells follicular helper, (d) macrophages M1, (e) plasma cells, (f) mast cells resting, (g) T cells CD8, (h) NK cells resting, (i) macrophages M0, and (j) macrophages M2. The correlation test was conducted using Pearson coefficient.

To delve into the association of TEX41 levels with the immune microenvironment, the proportion of tumor-infiltrating immune subsets was examined by the use of the CIBERSORT algorithm, and 21 kinds of immune cells in SKCM specimens were established (Figures 2(a) and 2(b)). We observed that T cells regulatory (Tregs) were positively associated with B cells naive. Correlation assays revealed that ten kinds of TICs were related to the expressions of TEX41 (Figures 3(a)–3(j)). Specifically, eight kinds of TICs were positively correlated with TEX41 expression, including dendritic cells resting (Figure 3(a)), T cells regulatory (Figure 3(b)), T cells follicular helper (Figure 3(c)), macrophages M1 (Figure 3(d)), plasma cells (Figure 3(e)), mast cells resting (Figure 3(f)), and T cells CD8 (Figure 3(g)). Three kinds of TICs were negatively related to TEX41 expressions, including NK cells resting (Figure 3(h)), macrophages M0 (Figure 3(i)), and macrophages M2 (Figure 3(j)). Our evidence suggested that TEX41 expressions exhibited a regulatory effect on the immune activity of tumor microenvironment.

### 3.3. GO and KEGG Pathway

**Figure 4:**
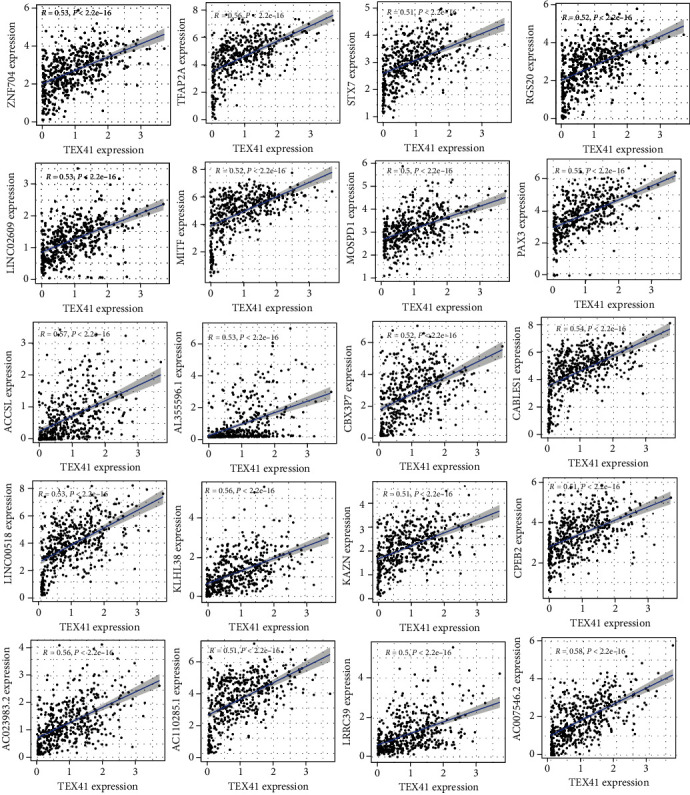
The coexpression genes of TEX41 were screened by correlation analysis.

**Figure 5:**
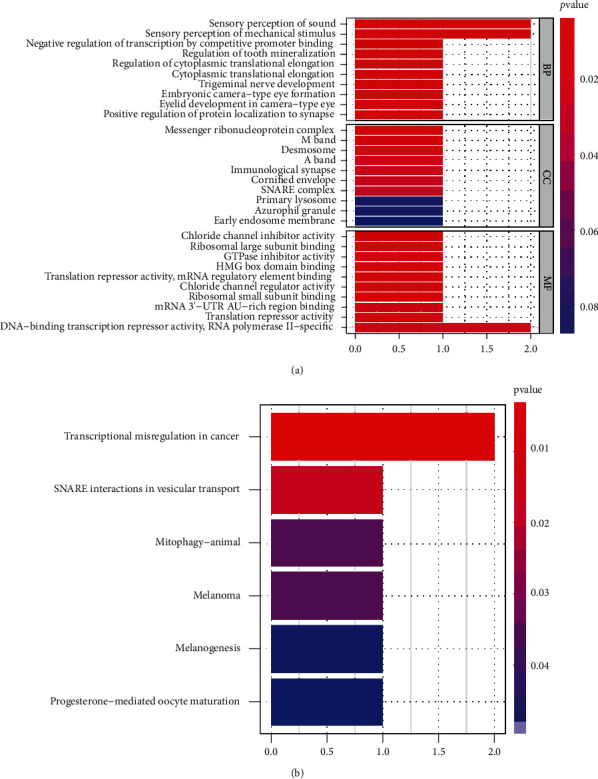
Significant (a) GO and (b) KEGG pathway analysis of the TEX41 coexpression genes.

Then, we performed correlation assays and found TEX41 expression was positively associated with 20 genes, including RGS20, ZNF704, STX7, AC007546.2, KLHL38, PAX3, CBX3P7, MITF, LINC02609, TFAP2A, AC023983.2, AC110285.1, MOSPD1, LINC00518, LRRC39, CPEB2, ACCSL, CABLES1, KAZN, and AL355596.1 (Figure 4). Then, we performed GO assays, and Figure 5(a) lists the top 10 of each aspect. The enriched biological process was involved in sensory perception of sound, sensory perception of mechanical stimulus, negative regulation of transcription by competitive promoter binding, regulation of tooth mineralization, regulation of cytoplasmic translational elongation, cytoplasmic translational elongation, trigeminal nerve development, eyelid development in camera-type eye, and positive regulation of protein localization to synapse. The enriched cellular components involved messenger ribonucleoprotein complex, M band, desmosome, A band, immunological synapse, cornified envelope, SNARE complex, primary lysosome, azurophil granule, and early endosome membrane. The enriched molecular function involved chloride channel inhibitor activity, ribosomal large subunit binding, GTPase inhibitor activity, HMG box domain binding, translation repressor activity, mRNA regulatory element binding, chloride channel regulator activity, ribosomal small subunit binding, mRNA 3′-UTR AU-rich region binding, translation repressor activity, and DNA-binding transcription repressor activity, RNA polymerase II-specific. There were 5 KEGG pathways related to the dysregulation of TEX41 expression (Figure 5(b)). The 5 pathways are transcriptional misregulation in cancer, SNARE interactions in vesicular transport, mitophagy-animal, melanoma, melanogenesis, and progesterone-mediated oocyte maturation.

## 4. Discussion

In recent years, the Food and Drug Administration has ratified the application of a number of antibodies targeting immune checkpoints for the treatment of SKCM because of their evident efficacies [24–26]. Unfortunately, the application of CD8+ T cells killing tumor cells is a very complex process, involving restimulation by tumor APC, overcoming local suppression, traffic to the tumor, T cell activation, antigen presentation, tumor antigen, and execution of tumor cells killing [27, 28]. If the above steps went out of order, the potential function of immunotherapy may be damaged, resulting in poor prognosis of SKCM patients. Thus, the identification of more sensitive and specific immunotherapeutic agents is necessary, which will help the preparation of patients which are suitable for immunotherapies [29, 30]. The diagnostic and prognostic biomarkers involved in the immune status would be tremendously valuable for the treatments of SKCM patients.

In this study, we identified a novel SKCM-related eRNA TEX41 which exhibited high expression in SKCM specimens via analyzing TCGA datasets. However, due to the small size of normal skin samples, more SKCM and nontumor specimens were needed to further determine the expressing pattern of TEX41 in SKCM. Moreover, we confirmed that high TEX41 was associated with poor prognosis of SKCM patients. Our findings suggested TEX41 as a potential prognostic biomarker for SKCM patients.

We firstly examined the composition of immune subsets because several immunotherapies have been constructed to modulate them. For example, T lymphocyte subsets, such as CD8+, acted as a positive indicator response to immunotherapies [31, 32]. We found that the patterns of immune cells are related to the outcome of SKCM patients. However, it is very hard for doctors to acquire the landscape of the infiltrating immune cells in clinical practice right now due to the expensive cost. The identification of sensitive biomarkers guiding the immune status of patients would be more feasible.

In recent years, several studies have reported the distinct dysregulation of TEX41 in several tumors, such as cervical cancer, head and neck squamous cell carcinoma, and leukemia [18–20]. However, the functional exploration of TEX41 in tumors was rarely reported. A previous study showed that TEX41 was highly expressed in lymphoblastic leukemia and TEX41 knockdown suppressed leukemic cell growth, suggesting TEX41 acted as a tumor promoter in leukemia [19]. In this study, we performed correlation analysis using TCGA datasets and demonstrated several genes which were positively associated with TEX41. Then, by the use of GO terms and KEGG pathways, we analyzed the possible effects of TEX41 in SKCM. The data revealed that TEX41 might be involved in programmed cell death, cancer metabolisms, and several pathways in tumors. However, more in vitro and in vivo assays were needed to demonstrate our findings.

## 5. Conclusion

In summary, increased TEX41 expression predicts an unfavorable prognosis. TEX41 expression is associated with the infiltration of various immune cells. TEX41 may act as a regulator in the tumor microenvironment of SKCM. TEX41 may represent a novel potential therapeutic target and prognostic marker for SKCM.

## Data Availability

The datasets analyzed during the current study are available from TCGA data repository (https://gdac.broadinstitute.org/) and GEPIA (http://gepia.cancer-pku.cn).
